# Serum amyloid a gene polymorphism and its association with lipid profile in Saudi females with osteoporosis

**DOI:** 10.12669/pjms.315.7981

**Published:** 2015

**Authors:** Azza M. Abdu-Allah, Shereen A. El Tarhouny, Hussam Hussein Baghdadi

**Affiliations:** 1 Azza M. Abdu-Allah MD. Menofyia University, Egypt. Department of Biochemistry and Molecular Medicine, Faculty of Medicine, Taibah University, Saudi Arabia; 2 Shereen A. El Tarhouny MD. Zagazig University, Egypt. Department of Biochemistry and Molecular Medicine, Faculty of Medicine, Taibah University, Saudi Arabia; 3 Hussam Hussein Baghdadi, PhD. Department of Biochemistry and Molecular Medicine, Faculty of Medicine, Taibah University, Saudi Arabia

**Keywords:** Osteoporosis, SAA1, Genotyping, Allel, Real time

## Abstract

**Background and Objective:**

Osteoporosis can be defined as a systemic skeletal disease characterized by low bone mass and micro architectural decline of bone tissue. Serum amyloid A (SAA) is a family of protein that increases up to 1,000-fold in blood during inflammation. In this study, we aimed to study the relationship between SAA1 gene polymorphism (rs12218) and lipid profile and osteoporosis.

**Methods:**

The study was performed on the female students of Taibah University in Al Medina, KSA during June 2014 to April 2015. According to BMD; osteoporosis group (138 students) and control group (128 students). All groups were subjected to; BMI, BMD, calcium, phosphorus, creatinine, lipid profile and SAA. Polymerase chain reaction and Real Time were done to determine the distribution of allele and genotype frequency of SAA (rs12218) C/T polymorphism.

**Results:**

This study shows that the TT genotype of rs12218 was more frequent in osteoporosis group than control group (P<0.001). Also, TT genotype and T allel was found to be associated with plasma total cholesterol, TG, LDLc, HDLc, Tscore, Zscore and SAA1 level in osteoporosis group (P=0.000, P=0.05, and P=0.000, P=0.000, P=0.01, P=0.02, P=0.000 respectively). The logistic regression analysis with and without lipid disorders in the osteoporosis group also show that the TT genotype of rs12218 still differed significantly between these two groups (P=0.001, OR=1.814, 95% CI: 0.719-4.577).

**Conclusion:**

The results of this study shows a significant association between TT genotype of rs12218 and both lipid level and osteoporosis in Saudi female population.

## INTRODUCTION

Osteoporosis can be defined as “a systemic skeletal disease characterized by low bone mass and micro architectural decline of bone tissue”.^1^ According to criteria of the World Health Organization (WHO) for diagnosis of osteoporosis, a densitometry evaluation-t score\-2.5 SD in the lumbar spine or the hip. If the annual bone loss after menopause is considered, around 50% women who have crossed 80 years of age are expected to be diagnosed with osteoporosis with few cases of fractures which are considered as the most severe clinical manifestations of this disease.^2^

A high prevalence of osteoporosis was reported in Saudi women in different areas in KSA and should be considered as a serious public health problem. A large scale multicenter, screening should be organized to identify the incidence of postmenopausal osteoporosis among Saudi women. Severity of bone loss should be evaluated by bone densitometry, by which patients who need treatment could be identified and also early diagnosis and follow up of those with osteopenia in order to introduce proper therapy and prevent future osteoporosis.^3^,^4^

Osteoporosis is significant risk factor for morbidity and mortality in older adults.^5^ It was reported that prevalence of osteoporosis increases with age, from 6% at 50 years to 50% after the age of 80. An estimated 50% of women and 20% of men above the age of 50 will have an osteoporosis-related fracture.^6^ The increasing prevalence of osteoporosis related fractures may increase the socioeconomic burdens because of the high cost of treatment.^7^

Serum amyloid A (SAA) is a family of protein that includes acute-phase and constitutive members. SAA has a multisegment structure of amphiphilic helix that is identical to helical apolipoprotein and is found bound to HDL, especially HDL3, in the blood.^8^

HDL particles bound to SAA are different from regular apoA-I-containing HDLs in their physicochemical properties and they may lead to abnormal functions. Replacement of apoA-I from HDL by SAA leads to an increase of HDL clearance and decrease in HDL blood level. A recent study, indicated that SAA, either lipid-bound or free, could compete with HDL binding to SR-BI and prevents cellular cholesteryl ester uptake from HDL.^9^,^10^

The SAA1 gene is located on the short (p) arm of chromosome 11 at position 15.1. The gene for SAA1 was considered as a candidate for osteoporosis because it is the gene encoding one important inflammatory factor; SAA.^11^

Alternate splicing results in multiple transcript variants that encode the same protein. Several studies have proved that rs12218 polymorphism in the SAA1 gene was associated with carotid atherosclerosis^12^ and peripheral arterial disease.^13^ However, the link between SAA gene polymorphisms and osteoporosis remain vague.^14^

In this study, our aim was study the relationship between SAA1 gene polymorphism (rs12218) and lipid profile and osteoporosis.

## METHODS

The study was performed on female students of Taibah University in Al Madinah, KSA during June 2014 to April 2015. The study was approved by the ethical committee of Taibah University. Written informed consent was obtained from all subjects prior to their inclusion into the study. Two hundred and sixty six students were involved in the study. After doing Bone mineral density for them, they were classified into two groups; osteoporosis group (138 students) and control group (128 students). Both osteoporosis and osteopenia females were gathered in one group which is osteoporosis group.

### Exclusion criteria

Students with delayed puberty, anorexia nervosa, primary or secondary amenorrhea, thyroid disease, chronic illness, bone or metabolic disorders and those taking medications which can affect the bone (antiepileptic, corticosteroids, antidepressants, vitamin D, calcium) were excluded by history taking and revising their medical files at Taibah Medical Unit. Patients with known chronic back pain and history of fractures were also excluded. Each participant was subjected to complete clinical examination, height and weight measurement. BMI was calculated (kg/m2).

### Bone mineral density measurement

was done on one-leg of all study participants, with the subject in the sitting position, using a water-based Achilles Express ultrasonometer ’’Lunar Achilles Express’’ QUS examination. The patient’s result is expressed as a T-score and Z-score as a percentage compared to the reference population. The diagnostic criteria for osteopenia/osteoporosis in the studied subjects was characterized according to WHO classification (1994) using T-score (>−1: normal, −1 to −2.49: osteopenia and −2.5 or less: osteoporosis). Low BMD (osteopenia and osteoporosis) was considered if the T score is of −1 or less.

### Sampling

Blood samples were drawn after an overnight fast under complete aseptic condition. Three ml of blood was placed in EDTA tubes for DNA extraction. The rest of blood was put in a plain tube for serum separation. The obtained serum was frozen immediately at −70°C for future analysis.

Serum calcium, phosphorous, creatinine, total cholesterol, triglyceride, and HDL-C concentrations were determined by Synchron *Cx9. LDL-C concentration was calculated according to the Friedewald formula. Serum Amyloid A1 was measured using solid-phase enzyme-linked immunosorbent assay (Hycult Biotech, Frontstraat 2a, 5405 PB Uden, the Netherlands).

### DNA Extraction

DNA extraction was done using QIAmp DNA blood kit (Qiagen, Valencia, CA) according to the manufacturer’s protocol. The purity of the DNA was determined by calculating the ratio of absorbance at 260/280 nm. All the purified samples were stored at -80°C until further analyses. The polymerase chain reaction (PCR) was used to detect the selected genetic polymorphisms.

### Detection of gene polymorphisms

Allelic variations were genotyped using the duplex quantitative TaqMan 5’ Allelic Discrimination Assay (Applied Biosystems, Foster City, CA) using established protocols as directed by the manufacturer. The genotyping assay for rs12218 is a custom design with ID number AHZAG95. Briefly, assay was performed in a final volume of 10 μl (including Taqman Genotyping Master Mix, 40x SNP Genotyping Assay Mix, DNase-free water, and 10ng DNA) in 96-well plates using the following amplification protocol: 95°C for 10 minutes followed by 50 cycles at 95°C for 15 seconds and 60°C for one minutes (annealing/extension). Fluorescence detection took place at 60°C. Non template controls were included in each run. The genotype call rate was over 99%. Duplicate genotyping of 10% of samples selected at random was performed for quality control. Assay was performed using StepOnePlus system and the automated sequence detection software (SDS) v2.3 was used for auto-calling (Applied Biosystems, Foster City, CA, USA).

### Statistical Analysis

The data were evaluated using SPSS *IBM Corp. Released 2012*. IBM SPSS Statistics for Windows, Version 21.0. Armonk, NY: IBM Corp. The continuous variables were not normally distributed based on the Shapiro-wilk test for normality. The mann-whitney U test was implemented for the comparison of the two groups. N and % values are provided. A p <0.05 was considered statistically significant.

## RESULTS

**Fig.1:**
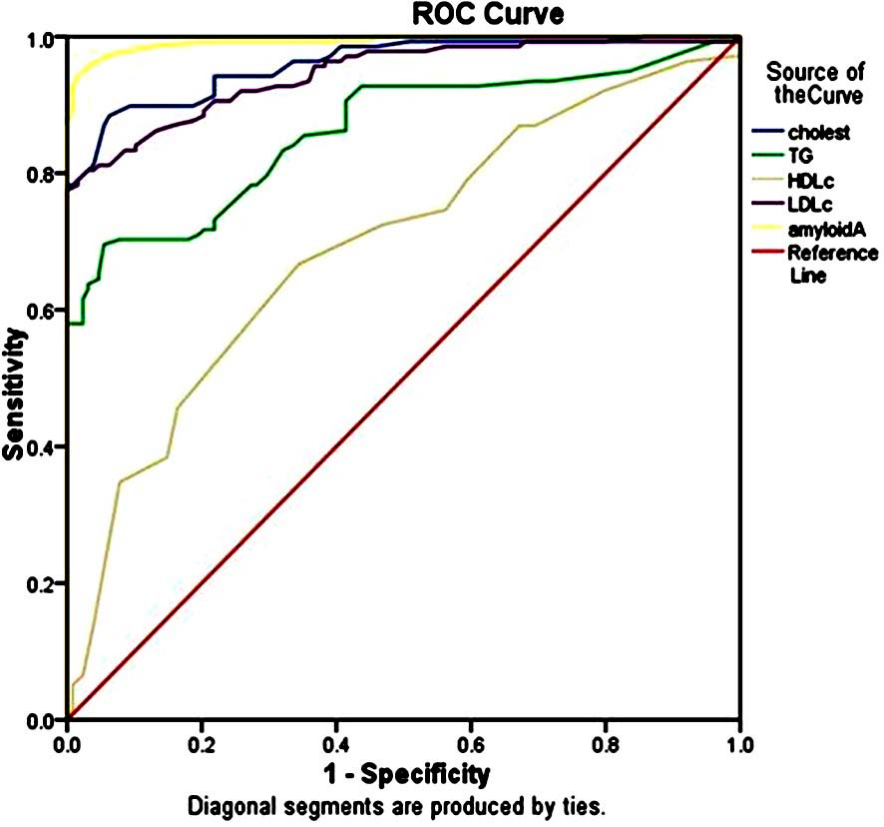
Roc curve for studies variable among Osteoporosis group (lipid profile, Amyloid A).

**Fig.2:**
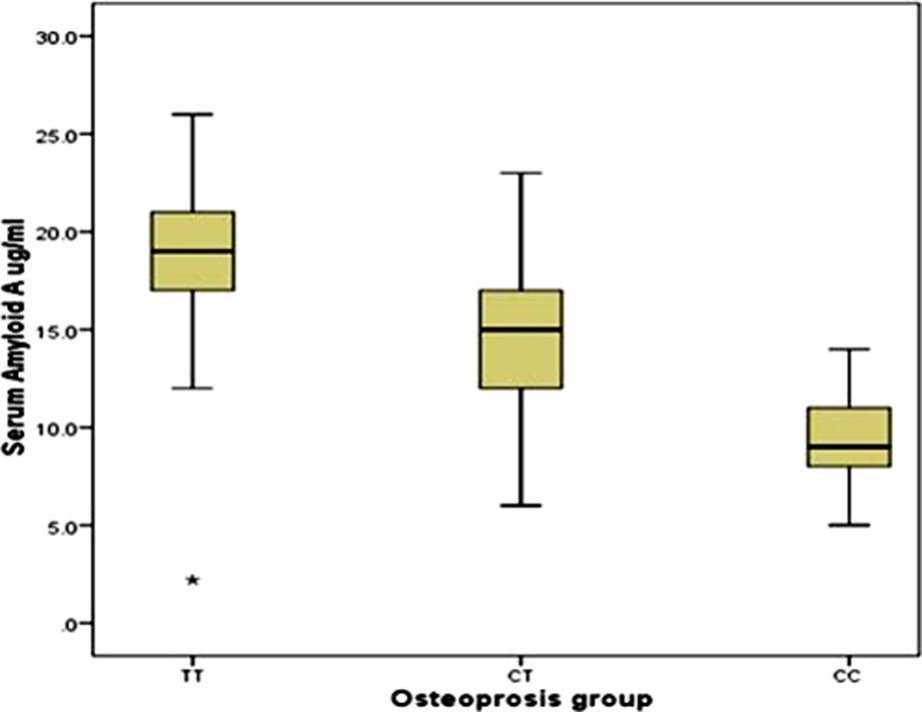
Shows relationship between serum amyloid A concentration and different genotypes of rs12218 (CC, CT, TT) in Osteoporosis group.

**Fig.3:**
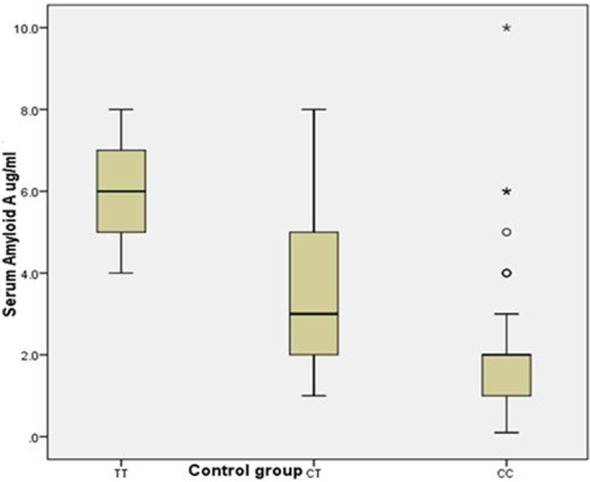
Shows relationship between serum amyloid A concentration and different genotypes of rs12218 (CC, CT, TT) in control group.

Table-I shows the clinical characteristics of the participants, Table-II shows the genotype and allele frequency among the studied groups, Table-III shows the relation between different rs12218 genotypes and lipid profile, T score, Z score, and serum amyloid A (SAA). Logistic regression analysis with and without lipid disorders in the osteoporosis group are shown in Table-IV. Fig.1 shows the ROC curve of lipid profile and SAA among osteoporosis group. Fig.2 shows the relationship between serum amyloid A concentration and different genotypes of rs12218 (CC, CT, TT) in Osteoporosis group. Fig.3 shows the relationship between serum amyloid A concentration and different genotypes of rs12218 (CC, CT, TT) in control group.

**Table-I:** Clinical characteristics of the participants.

Parameters	Osteoporosis group (N=138) Mean ± SD	Control group (N=128) Mean ± SD	P value
Age (years)	22.15 ± 1.89	21.76 ± 2.01	0.10
BMI (Kg/m^2)^	20.83 ± 1.87	21.23 ± 2.13	0.09
T score	-2.46 ± 0.47	1.10 ± 0.98	0.000*
Z score	-2.54 ± 0.45	1.16 ± 0.98	0.000*
Ca (mg/dl)	7.07 ± 0.52	9.16 ± 0.39	0.03*
Ph (mg/dl)	4.82 ±0.85	4.16 ± 0.55	0.21
Creatinine (mg/dl)	0.72 ± 0.11	0.71 ± 0.14	0.15
Cholesterol (mg/dl)	233.28 ± 31.27	170.83 ± 15.06	0.000*
Triacylglycerol (mg/dl)	127.88 ± 31.30	86.82 ±12.62	0.000*
HDLc (mg/dl)	48.98 ±3.95	46.52 ± 3.64	0.05*
LDLc (mg/dl)	157.39 ± 27.24	107.15 ± 16.24	0.000*
Serum Amyloid A (ug/ml)	16.15 ± 4.84	2.85 ± 2.01	0.000*

**Table-II:** Genotype and allele frequency among the studied groups.

	Genotypes			Allele		
	CC	CT	TT	C	T	P value
Osteoporosis group (n=138)	18 (13.0)	53 (38.4)	67 (48.6)	89	187	<0.001*
Control group (n=128)	66 (51.6)	56 (43.8)	6 (4.7)	188	68	

**Table-III:** The relation between different rs12218 genotypes and lipid profile, T score, Z score, and serum amyloid A (SAA).

Parameter	Genotypes	Osteoporosis group			Control group		
		N	(M±SD)	p value	N	(M±SD)	p value
Cholesterol (mg/dl)	CC	18	209.2±23.1	0.000*	66	172.1±11.2	0.04*
	CT	53	221.8±29.21	56		179.9±13.39	
	TT	67	248.8±26.64	6		181.33±10.01	
TG (mg/dl)	CC	18	109.2±25.90	0.05*	66	82.5±9.23	0.24
	CT	53	120.09±32.18	56		83.83±10.38	
	TT	67	139.07±27.80	6		93.39±14.11	
HDLc (mg/dl)	CC	18	44.55±3.56	0.000*	66	44.83±3.48	0.05*
	CT	53	47.86±3.77	56		44.48±3.25	
	TT	67	51.06±2.69	6		48.40±2.93	
LDLc (mg/dl)	CC	18	142.7±19.11	0.000*	66	107.2±12.16	0.17
	CT	53	147.52±29.29	56		117.6±13.27	
	TT	67	169.14±22.21	6		119.33±11.75	
Z score	CC	18	-2.10±0.42	0.01*	66	1.27±0.93	0.03*
	CT	53	-2.37±0.48	56		1.26±1.07	
	TT	67	-2.80±0.25	6		1.02±1.04	
T score	CC	18	-2.16±0.44	0.02*	66	1.23±0.91	0.05*
	CT	53	-2.38±0.49	56		1.25±1.04	
	TT	67	-2.72±0.26	6		0.98±1.07	
Serum Amyloid A (ug/ml)	CC	18	9.22±2.31	0.000*	66	2.01±1.70	0.05*
	CT	53	14.79±3.62		56	3.50±1.83	
	TT	67	19.09±3.69		6	6.00±1.41	

**Table-IV:** Logistic regression analysis in the osteoporosis group.

Variable	B	SE	Wald	df	Sig.	OR (95% CI)
Age	0.078	0.107	0.526	1	0.468	1.08 (.876 - 1.335
BMI	-0.016	0.102	0.024	1	0.878	0.984 (.806 - 1.203
Calcium	-1.281	0.528	5.881	1	0.015*	0.278 (.099 -0.782)
Phosphurs	0.118	0.303	0.151	1	0.697	1.125 (0.622 - 2.036)
Creatinine	1.602	1.718	0.870	1	0.351	4.965 (0.171 -144.0)
TT	0.596	0.472	1.590	1	0.001*	1.814 (0.719 - 4.577)
CC	1.018	0.719	2.004	1	0.157	2.766 (0.676 - 11.317)
SAA	0.156	0.055	8.110	1	0.004*	1.169 (1.050 - 1.302)
Constant	3.213	6.354	0.256	1	0.613	-

## DISCUSSION

This study was done on the young female students of Taibah University as we noticed that there is a large number of them expressing osteopenia and osteoporosis. We assumed that the feeding behavior and their life style comprise one of the major causes of osteoporosis. They are always indoors without sun exposure, consuming large amounts of gaseous drinks and there is no exercises practiced (taking from their questionnaire). So we needed to study genetic susceptibility and their genetic background.

In the current study, we found that genotypic variation in the SAA1 genes is associated with both Total Cholesterol, TG, HDLc, LDLc, Tscore, Zscore and SAA levels in osteoporosis and control group of the Saudi women population.

Osteoporosis is a dormant disease expressed several times by low bone density till a fracture happens. It is called ‘silent epidemic as it is a rising problem and many patients are asymptomatic.^15^ Osteoporosis leads to porous bones which is characterized by low bone mass and structural worsening of bone tissue, ending by increasing bone fragility and an increased risk for fractures of the hip, spine, and wrist.^16^

Osteoporosis is classified as primary or secondary depending on the pathogenesis of the disease. Primary osteoporosis occurs in males and females and occurs as a consequence of aging and decreased gonadal functions. So it occurs usually after menopause in women and later in life in men.^17^ Secondary osteoporosis can occur from endocrine/metabolic disorders, nutritional conditions, medications, and immobilization. These conditions affect the osteoclast count and activity associated with bone wear and tear.^14^

There was a great interest of SAA gene polymorphism as it encodes a very important cytokines SAA. Serum amyloid A (SAA) is a protein family that is divided into acute-phase and constitutive members, both of which are synthesized mainly in the liver and the former is in reaction to the inflammatory status.^9^ Constitutive SAA which is SAA4 is present only in human and mouse. Acute-phase SAA which are SAA1 and SAA2 is present in all of the vertebrates investigated and can raise up to1,000-fold increase in human plasma during inflammation.^9^ A relationship between the SAA1 gene polymorphism and cardiovascular diseases was found in another study concerning cardiovascular disease as a chronic inflammation.^18^,^19^

In our current study, the TT genotype of rs12218 significantly differed between osteoporosis patients and control participants, indicating that the risk of osteoporosis is increased in participants with the T allele of rs12218 and also the Logistic regression analysis showed that TT genotype distribution of rs12218 significantly differed between the osteoporosis patients and the control participants. These results came along with a study done by Zheng et al.^14^

Regarding our study, we found that variation in SAA gene (rs12218) was significantly associated with the plasma level of total cholesterol, TG, HDLc and LDLc levels in the osteoporosis patients, these results comes on line with Zheng et al.^14^ & Xie et al.^20^

The human SAA gene is found on the short arm of chromosome 11, restricted to band p15.1, consists of four related genes, SAA1-4, within a 150-kb region.^10^ Only the SAA1 and SAA2 genes encode acute-phase SAAs (SAA1 and SAA2 proteins), so many studies emphasize on them. Carty et al.^21^ found a relationship between SAA1 and SAA2 gene polymorphisms and carotid intima-media thickness (cIMT), HDL-C, and total CVD. Many studies showed that rs12218 in the SAA1 gene was associated with peripheral arterial disease^11^ and carotid atherosclerosis.^13^

SAA is found associated with HDL, especially HDL3, in blood.^22^ It was found that SAA is an analog of helical apolipoprotein and is fully capable of forming new HDL particles with cellular lipid in the presence of ABCA1 or ABCA7 in the cell membrane.^23^ It was found that HDLs associated with SAA have different physicochemical characteristics than the normal types generated with apoA-I.^24^

In plasma, SAA is associated with HDL and, during severe inflammation, can form 80% of its apo-protein composition. The detached apoA-I is rapidly removed from blood by the kidneys and liver. This process is accompanied by a rapid decrease in apoA-I gene expression in blood during inflammation. HDL plays an important role in cholesterol transport from extrahepatic peripheral cells to the liver as an important mechanism of cholesterol catabolism. This mechanism is important guard against atherosclerosis. It was found that SAA stimulate cellular lipid release both in ABCA1-dependent and -independent mechanisms.^22^,^25^

## CONCLUSIONS

SAA rs12218 gene polymorphism was associated with osteoporosis in Saudi females and this association may be related to the lipid disorder resulting from the SAA gene polymorphisms especially TT genotype. We also assumed that the nutritional behavior, lack of immobilization and deficient exposure to sun accelerate the rate of development of osteopenia in genetically susceptible females at younger age unexpectedly.
